# Small cell variant of chromophobe renal cell carcinoma: Clinicopathologic and molecular-genetic analysis of 10 cases

**DOI:** 10.17305/bjbms.2021.6935

**Published:** 2022-03-10

**Authors:** Joanna Rogala, Fumiyoshi Kojima, Reza Alaghehbandan, Nikola Ptakova, Ana Bravc, Stela Bulimbasic, Delia M. Perez Montiel, Maryna Slisarenko, Leila Ali, Levente Kuthi, Kristýna Pivovarčíková, Květoslava Michalová, Boris Bartovic, Adriena Bartos Vesela, Olga Dolejsova, Michal Michal, Ondrej Hes

**Affiliations:** 1 Department of Pathology, Charles University in Prague, Faculty of Medicine in Plzeň, Pilsen, Czech Republic; 2 Department of Pathology “Hist-Med,” Regional Specialized Hospital, Wroclaw, Poland; 3 Department of Human Pathology, Wakayama Medical University, Wakayama, Japan; 4 Department of Pathology, Faculty of Medicine, University of British Columbia, Royal Columbian Hospital, Vancouver, Canada; 5 Department of Biology and Medical Genetics, 2^nd^ Faculty of Medicine, Charles University in Prague and Motol University Hospital, Prague, Czech Republic; 6 Department of Pathology, General Hospital, Slovenj Gradec, Slovenia; 7 Department of Pathology, University Hospital Centre Zagreb, Zagreb, Croatia; 8 Department of Pathology, Institute Nacional de Cancerologia, Mexico City, Mexico; 9 Department of Pathology, CSD Laboratory, Kiev, Ukraine; 10 Department of Pathology, “Carol Davila” University of Medicine and Pharmacy, Bucharest, Romania; 11 Department of Pathology, University of Szeged, Szeged, Hungary; 12 Departmnet of Pathology, Cytopathos, Bratislava, Slovakia; 13 Department of Urology, Charles University in Prague, Faculty of Medicine in Plzeň, Pilsen, Czech Republic

**Keywords:** Kidney, chromophobe renal cell carcinoma, small-cell variant

## Abstract

The morphologic diversity of chromophobe renal cell carcinoma (ChRCC) is well-known. Aside from typical morphology, pigmented adenomatoid, multicystic, and papillary patterns have been described. Ten cases of CHRCC composed of small-cell population in various percentages were analyzed, using morphologic parameters, immunohistochemistry, and next-generation sequencing testing. Patients were five males and five females, with age ranging from 40 to 78 years. The size of tumors ranged from 2.2 cm to 11 cm (mean 5.17 cm). Small-cell component comprised 10 to 80% of the tumor volume, while the remaining was formed by cells with classic ChRCC morphology. The immunohistochemical profile of the small-cell component was consistent with typical ChRCC immunophenotype, with CD117 and CK7 positivity. Neuroendocrine markers were negative. Mutations of 13 genes were found: *DCIER1, FGFR3, JAK3, SUFO, FAM46C, FANCG, MET, PLCG2, APC, POLE, EPICAM, MUTYH*, and *AR*. However, only the *PLCG2* mutation is considered pathogenic. The small-cell variant of ChRCC further highlights and expands on existing morphologic heterogeneity spectrum. Recognition of small-cell variant of CHRCC is not problematic in tumors, where the “classic” CHRCC component is present. However, in limited material (i.e., core biopsy), this may present a diagnostic challenge. Based on the limited follow-up data available, it appears that the small-cell tumor component had no impact on prognosis, since there was no aggressive behavior documented. Awareness of this unusual pattern and applying additional sections to find classic morphology of ChRCC, as well as excluding neuroendocrine nature by immunohistochemistry, may help resolve difficult cases.

## INTRODUCTION

Several morphologic variations of chromophobe renal cell carcinoma (ChRCC) have been reported since Thoenes and Storkel [1,2] first described it. Cases with morphology that differs from the typical solid-alveolar architecture seen in classic or eosinophilic ChRCC have been well-documented in the literature, including adenomatoid pigmented ChRCC, ChRCC with neuroendocrine differentiation (or with neuroendocrine-like differentiation), oncocytic ChRCC, multicystic ChRCC, and ChRCC with papillary architecture [3-9].

The small-cell variant of renal oncocytoma (RO) is a well-defined morphologic subtype of a common renal tumor [10-12]. In addition, ChRCC and RO are thought to be closely related tumors derived from the intercalated cells. However, small-cell variant of ChRCC has not been described. We selected a group of ChRCCs with a small-cell component forming from 10 to 80% of the tumor volume. Clinicopathologic, morphologic, immunohistochemical, and molecular genetic analysis of 10 cases were performed.

## MATERIALS AND METHODS

The database of Tumor Registry in Plzen was searched for keywords: Kidney; oncocytoma; and chromophobe. A total number of 2067 tumors were retrieved. All ChRCCs with “classic” morphology, as well as the eosinophilic variants, were excluded. Since no case from the RO cohort was reclassified as a small-cell variant of ChRCC, all ROs were excluded. All ChRCC with so-called variant histology were re-evaluated. We particularly focused on cases with true neuroendocrine differentiation, which were excluded after the initial immunohistochemical staining for synaptophysin, chromogranin, and CD56 (see later for details). Three cases with strong focal CD56 positivity in the small-cell tumor component were also eliminated from the study. Out of 1092 ChRCC and 975 RO cases from the Plzen Tumor Registry, 13 cases were found to be suitable. For the final selection, a 10% cutoff for the small-cell component was applied. Ultimately, 10 cases were enrolled in the study. Each participating institution provided clinical data and follow-up information. None of the cases included in the study had ever been reported before. Tissues for microscopic examination were formalin fixed and paraffin embedded using standard procedure. Two to 4 μm thick sections were cut and stained for hematoxylin and eosin. For each case, 1-13 paraffin blocks were available. All of the tumors were independently reviewed by three pathologists (JR, FK, and OH).

### Immunohistochemistry

The immunohistochemical study was performed using a Ventana Benchmark XT automated immunostainer (Ventana Medical System, Inc., Tucson, AZ, USA) on formalin-fixed paraffin-embedded (FFPE) tissue. The primary antibodies used were as follows: CK7 (OV-TL12/30, monoclonal, DakoCytomation, Glostrup, Denmark, 1:200), cytokeratin 20 (M7019, monoclonal; Dako; 1:100), vimentin (D9, monoclonal, NeoMarkers, Westinghouse, CA, 1:1000), CD56 (1B6, monoclonal, Leica Biosystems, Newcastle, UK, 1:100), synaptophysin (polyclonal, LabVision, Fremont, CA, 1:350), chromogranin A (monoclonal, DAK-A3, DakoCytomation, 1:600), c-kit (CD117, polyclonal, DakoCytomation, 1:300), TTF1 (monoclonal, SPT24, Ventana, 1:400), GATA3 (monoclonal, L50-823, Biocare Medical, Concord, CA, 1:100), NKX3.1 (polyclonal, Biocare Medical, 1:50), FLI 1 (monoclonal, MRQ-1, Cell Marque, Rocklin, CA, 1:50), CD99 (monoclonal, HO36-1.1, Neo Markers, Rockford, IL, 1:200), WT1 (monoclonal, 6F-H2, DAKO, 1:50), and napsin (polyclonal, Ventana, RTU), Ki-67 (monoclonal, MIB-1, DAKO, 1:400). The primary antibodies were visualized using a supersensitive streptavidin-biotin-peroxidase complex (BioGenex). Internal biotin was blocked by the standard protocol used by Ventana Benchmark XT Automated Stainer (hydrogen peroxide based).

Appropriate positive and negative controls were employed. The slides were evaluated as follows: (−) Negative; (±) <10% positive cells; (+) 10-25% positive cells; (++) >25-50% positive cells; (+++) >50-75% positive cells; and (++++) >75% of positive cells.

### Molecular genetic methods

Mutation analysis was performed using the TruSight Oncology 500 assay (Illumina, San Diego, CA). Total nucleic acid was extracted using the FFPE DNA kit (automated on RSC 48 Instrument, Promega, Madison, Wisconsin, USA). Purified DNA was quantified using the Qubit Broad Range DNA. The quality of DNA was assessed using the FFPE QC kit (Illumina), and DNA samples having Cq<5 were used for further analysis. After the DNA enzymatic fragmentation with KAPA Frag Kit (KAPA Biosystems, Washington, MA), DNA libraries were generated using the TruSight Oncology 500 assay (Illumina), according to the manufacturer’s protocol.

Sequencing was performed using the NextSeq 550 sequencer (Illumina) following the manufacturer’s guidelines. Data analysis (DNA variant filtering and annotation) was performed using the Omnomics Next-generation sequencing (NGS) analysis software (Euformatics, Finland). The custom variant filter was set up including only non-synonymous variants with coding consequences, read depth greater than 50. Benign variants according to the ClinVar database were excluded as well [13]. The remaining subset of variants was examined visually, and any apparent artefactual variants were excluded.

### Ethical statement

The study was performed in accordance with the Declaration of Helsinki. Ethics committee approval was not required by Charles University and University Hospital Plzen.

## RESULTS

Table 1 summarizes the basic clinicopathologic data. The patients ranged in age from 40 to 78 years old (median 58.5 years; mean 58.5 years), with five males and five females. According to UICC 2017, four patients presented with pT1a stage, one with pT1b, one with pT2a, one with pT2b, and three with pT3a. Follow-up was provided in nine cases, ranging from 24 to 73 months (mean 50.75 months; median 48 months). Eight of the patients were alive with no evidence of disease progression. One patient was diagnosed with concurrent pancreatic carcinoma at stage pT3a and died due to widespread metastatic disease following surgery and treatment.

**TABLE 1:**
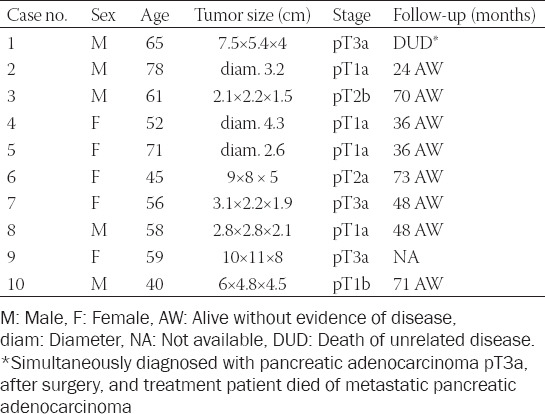
Basic clinicopathologic data of ChRCC with small-cell morphology

Tumor size spanned from 2.2 cm to 11 cm in the greatest dimension (mean 5.17 cm). Macroscopically, all lesions were well-demarcated and non-capsulated. On cut section, the tumorous parenchyma was orange-yellow to brownish in color, homogeneous, with no grossly visible necrosis. Morphologic features of the tumors are summarized in Table 2.

**TABLE 2:**
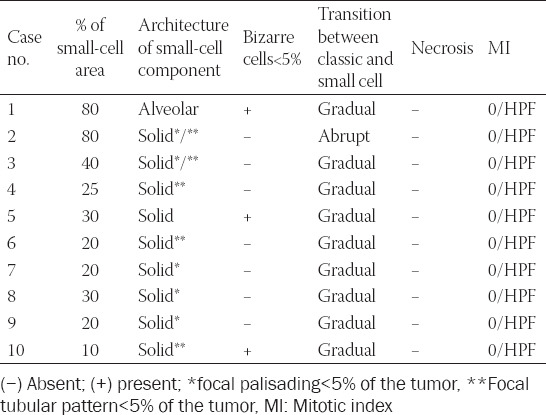
Morphologic parameters

**FIGURE 1:**
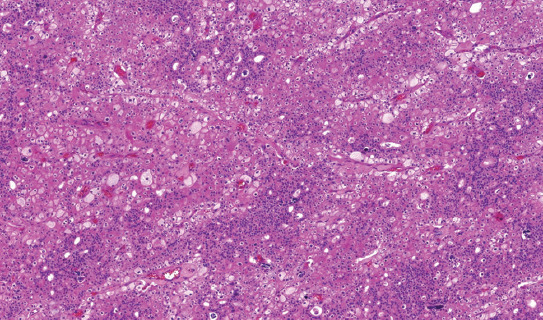
The distribution of the small-cell component was multifocal, with a gradual transition from classic ChRCC to the small-cell area in the majority of cases.

**FIGURE 2:**
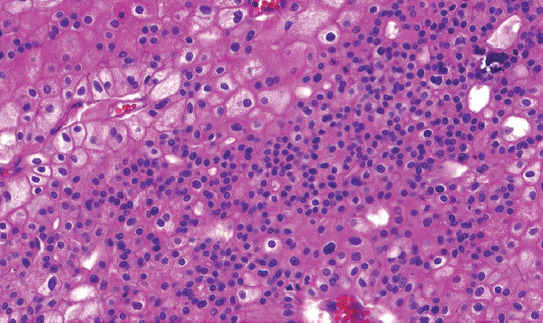
Classic chromophobe cells were intermingled among a dense population of small-cell component.

**FIGURE 3:**
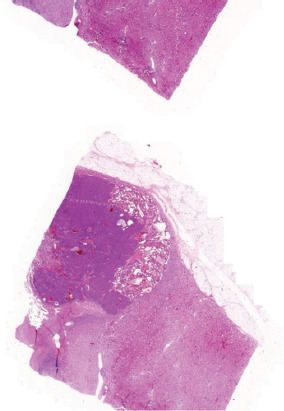
Case where the both components were sharply demarcated without transitional zone between both cell types.

**FIGURE 4:**
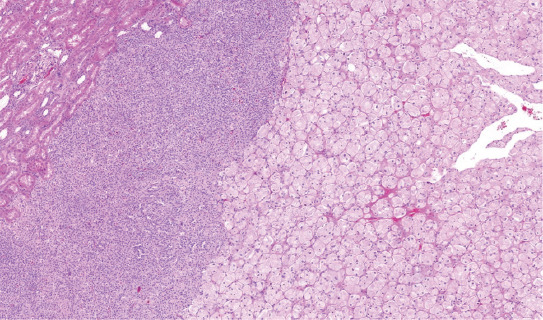
The architecture was solid in some cases.

Microscopically, all cases had “classic chromophobe” morphology, at least focally. The extent of small-cell component ranged from 10% to 80% of the tumor volume. The distribution of the small-cell component was multifocal with a gradual transition from classic ChRCC to the small cell area (Figures 1 and 2). In one case (case 2), both components were sharply demarcated (Figure 3). The architecture in a majority of the cases was predominantly solid (Figure 4), with small foci, nested, tubular, or palisaded arrangement in small-cell component and solid alveolar in a classic component.

**FIGURE 5:**
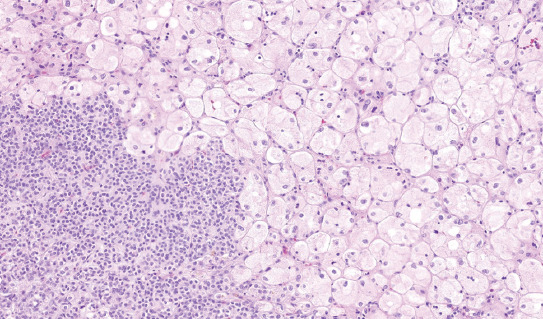
Effect of TDF and TDF-AgNPs on the prefrontal cortex pyramidal cell. The cells of classic component were typical with voluminous cytoplasm and raisinoid nuclei.

**FIGURE 6:**
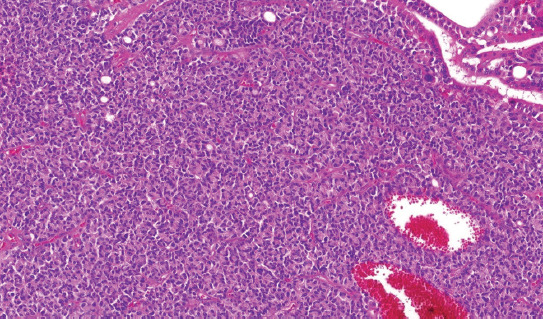
The cells in the small-cell component showed scant cytoplasm and round to oval nuclei. Mitotic activity was absent.

The cells of the classic component were typical, large, with voluminous cytoplasm and raisinoid nuclei, accompanied by smaller, eosinophilic cells with perinuclear clearing and occasional nuclei with irregular contours (Figure 5). Cells in the small-cell component showed scant cytoplasm, round to oval, and frequently overlapping nuclei with non-conspicuous nucleoli (Figure 6). No nuclear grooves or coffee bean patterns were documented. There were no nuclear grooves or coffee bean patterns. In both large and small-cell components, no mitotic figures were found.

**FIGURE 7:**
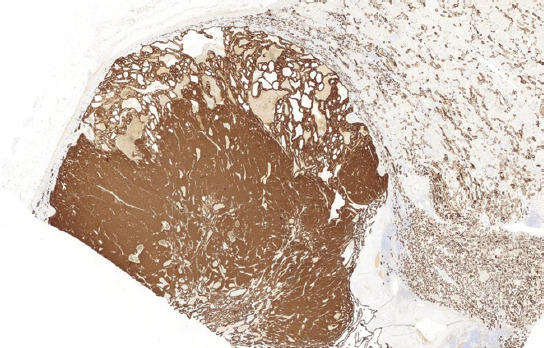
Tumors were CK7 positive in both components.

**FIGURE 8:**
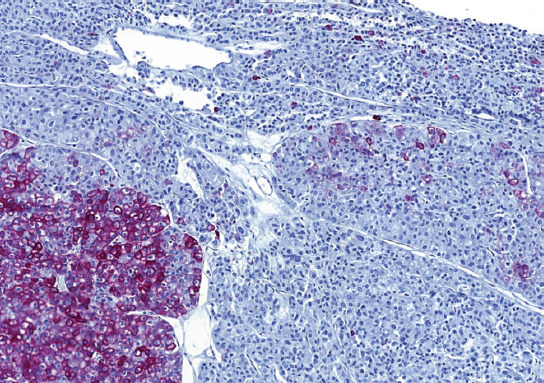
In case 9, small-cell component displayed a patchy pattern of reactivity with CK 7, superficially resembled reactivity of renal oncocytoma.

**FIGURE 9:**
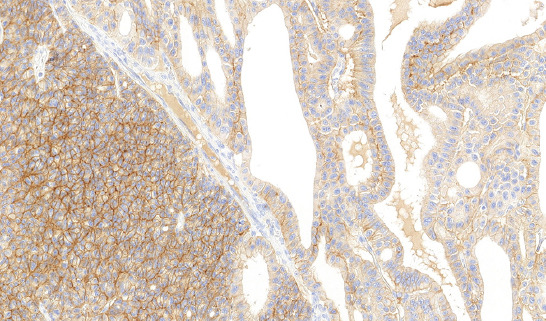
CD117 was positive in the vast majority of cases in diffuse membranous pattern.

In three cases, foci of bizarre cells with large, hyperchromatic nuclei similar to those frequently observed in oncocytoma (so-called polyploid cells) were present. None of the cases showed sarcomatoid transformation or necrosis. Results of immunohistochemical examination are summarized in Table 3A, 3B. Immunohistochemically, CK 7 staining pattern in small cell areas was almost identical to the staining pattern in classic ChRCC areas (Figure 7). In one case (case 9), the classic component of ChRCC showed diffuse, mosaic positivity, whereas the small-cell component showed a focal, oncocytoma-like pattern of staining (Figure 8). On cell membranes, CD117 was mostly diffusely positive, with weak to moderate intensity in both components (Figure 9). In one case (case 9), CD117 showed positive staining in the classic ChRCC component only. In both the classic and small-cell components, all cases were negative for synaptophysin and chromogranin. CD56 expressed focal to patchy, very weak positivity in large cells of the classic component in four cases, which was considered non-specific. FLI 1 was positive in one case (case 1) in both the classic and small-cell component. CK 20, GATA3, NKX 3.1, TTF1, napsin A, WT 1, and CD99 were negative in all cases. Ki-67 positivity ranged from 2 to 20 cells per HPF in both components. NGS analysis was successful in five cases. Results are summarized in Table 4.

**TABLE 3A:**
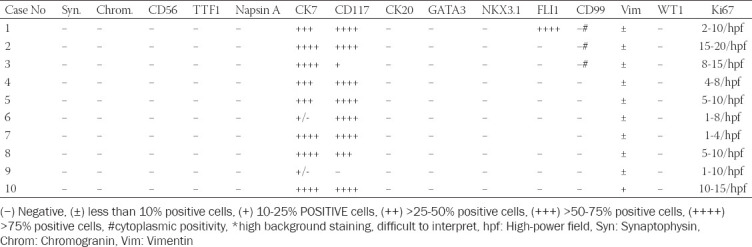
Results of immunohistochemical examination of small cell ChRCC component

**TABLE 3B:**
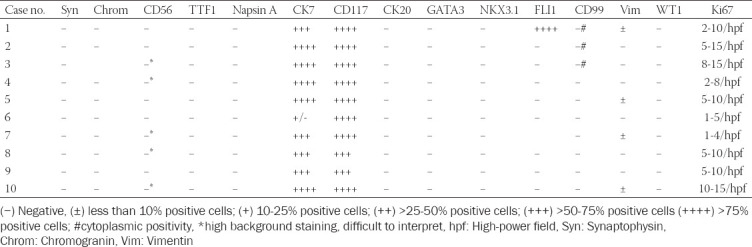
Results of immunohistochemical examination of classic ChRCC component

**TABLE 4:**
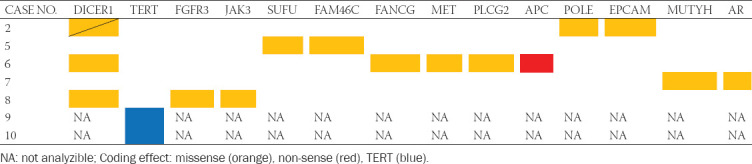
Results of next-generation sequencing

Mutations of 13 genes were found, namely, *DCIER1, FGFR3, JAK3, SUFO, FAM46C, FANCG, MET, PLCG2, APC, POLE, EPICAM, MUTYH*, and *AR*. However, only the *PLCG2* mutation is listed as pathogenic. No mutations of *FLCN, VHL, SDH, TSC1, TSC2*, and *MTOR* were documented. Seven cases were suitable for *TERT* hot spot analysis. Two tumors carried *TERT* mutation in position 228 (chr5:1295228 C>T).

## DISCUSSION

ChRCC and RO are considered tumors derived from so-called intercalated cells of collecting ducts [14,15]. In addition to a common cell of origin, they share several morphologic features. Both tumor types are usually located within the renal cortex, both well-circumscribed, yet non-encapsulated. In the gross section, both RO and ChRCC are predominantly brown, sometimes with a scar that is centrally located in RO. However, the central scar is not specific for RO and might be found in other renal tumors as well. Histologically, both tumors are composed of oncocytic cells, although the morphologic details and immunohistochemical features may differ. Because of these similarities, several researchers hypothesized that RO might be a potential “chromophobe adenoma” that could progress to ChRCC [16-18] in a manner similar to the adenoma-adenocarcinoma sequence in the colorectal cancer [19]. However, other authors, including the authors of this study disagree [20].

The small-cell variant of RO was first described in 2001 [10,21], although the existence of small oncocytic cells, so-called oncoblasts, was documented and discussed much earlier [22]. Several papers published afterward [11,23,24] further defined the small cell variant of RO.

ChRCC, in its classic form, is described as a solid-alveolar tumor composed of large leaf-like cells and smaller oncocytic cells. Several morphologic variants, which differed from classic morphology, were subsequently described in the literature. As the name indicates, the architecture of the adenomatoid microcystic pigmented variant comprises microcystic, cribriform areas mixed with conventional ChRCC pattern. Adenomatous structures lined by small cylindrical cells with basally located nuclei constitute a second component [4].

Multicystic ChRCC is composed either of variable-sized cysts or compressed cysts and tubules with slit-like spaces. The cellular lining is made up entirely of eosinophilic cells or a mixed population of eosinophilic and pale cells. It is likely that the two aforementioned variants represent a spectrum of one morphologic subtype in which the adenomatoid pattern progressively transforms to areas with microcystic architecture. Lipochrome pigment accumulation is constantly present in the former, whereas it was noted in <50% of cases in the latter group of tumors [8]. Within the ChRCC spectrum, ChRCC with papillary architecture is quite rare. However, it has been described in the literature [25]. Such a pattern was present focally. The cytologic features, on the other hand, followed a characteristic dual population of leaf-like and small eosinophilic cells. Foam cells were not present. Recently, series of CHRCC with prominent papillary architecture has been published [9]. The extent of papillary component in tumors ranged from 15 to 100% of the tumor volume. The cytologic characteristics were typical.

ChRCC with morphology similar to neuroendocrine tumors, namely, with trabecular/palisading/cribriform pattern was also documented. Among them were the CHRCC with true neuroendocrine differentiation, confirmed by positive staining for synaptophysin, chromogranin or CD 56 [5,7].

Tumors that showed similar architecture and cytologic features, but without positive neuroendocrine immunohistochemical staining were labeled as ChRCC with neuroendocrine-like features [7].

ChRCC with neuroendocrine differentiation and neuroendocrine-like features can be remarkably similar to the small cell variant of ChRCC. To rule out cases with true neuroendocrine differentiation, we employed three different neuroendocrine markers (synaptophysin, chromogranin, and CD56).

In the present study, the small-cell tumor population was uniform, showing mostly scant cytoplasm, arranged predominantly in solid, relatively compact areas or sheets. Only focal palisading or tubular structures were seen. Such patterns were located in transition zones between small-cell and classic ChRCC components, always comprising <5% of the small-cell component volume.

There is another parallel between the small cell oncocytoma and the small-cell variant of ChRCC. In ChRCC with an adenomatoid pattern, groups of small cells are located on the edges of adenomatoid structures or on the edges of fibrotic, scar-like foci. A similar phenomenon has been well-documented in a small cell variant of RO [11]. In classic RO, however, groups of small oncocytes are frequently found in identical location. Pseudorosettes or ribbon-like patterns were not seen in the small-cell ChRCC variant. The presence of such structures is an interesting phenomenon in the context of differential diagnosis. In a series of small-cell variants of RO [11], pseudorosettes with a PAS-positive central core were described. However, we do not believe such structures can be used as a differential diagnostic feature.

Immunohistochemical profiles of our cases were compatible with the classic variant of ChRCC, mostly showing strong, diffuse, or focal positivity for CK 7, along with diffuse or focal, weak to moderate positivity for CD117 in both tumor components. However, one case (case 9) was exceptional: The small-cell component expressed an oncocytoma-like CK7 staining pattern with diffuse, mosaic positivity in the classic part, whereas CD117 was positive solely in the classic part.

Focal weak positivity for CD56 was considered non-specific, and other neuroendocrine markers were negative. Interestingly, there was a strong positive immunohistochemical reaction for FLI1, which was present in both components in case 1. Unfortunately, the case was not suitable for molecular genetic analysis due to the low quality of DNA/RNA, but the morphology supported the diagnosis of ChRCC.

In cases with overlapping features between ChRCC, as well as cases with worrisome clinical features, association with the syndromic disease should be considered. According to clinical reports, we have no evidence of syndromic disease within our cohort. Furthermore, NGS was used to screen molecular profiles of our cases. Only five tumors were suitable for a complete NGS analysis. We were unable to document any genetic alteration linked to syndromic diseases. *FLCN*, *VHL*, and/or *SDH* gene mutations were not detected. The significance of the only pathogenic mutation of *PLCG2* gene found in our cohort remains unclear.

Renal tumors with mTOR pathway abnormalities were documented recently. Some of these newly recognized subtypes are characterized by an eosinophilic/oncocytic or chromophobe-like morphology [26]. Among them are eosinophilic solid and cystic RCC (ESC RCC), eosinophilic vacuolated tumor (EVT), and a low-grade oncocytic tumor, which can have eosinophilic/oncocytic or chromophobe-like morphology. Therefore, these tumors should be considered in the differential diagnosis of small-cell ChRCC variant. There were no morphologic features of the above-mentioned entities in our cases, and no mutations in the mTOR pathway genes were detected. However, one of our cases showed overlapping immunophenotype with EVT with positivity for CD117 and CK7 negative/focally positive.

The grading and biological behavior of ChRCC is notoriously inconsistent. Fuhrmans’ grading system, classic ISUP/WHO modification of Fuhrmans’ system [12], and even grading system proposed by Paner *et al*. are all practically not applicable [27,28]. Sarcomatoid transformation and/or necrosis were the only morphologic factors significantly associated with poor prognosis in a multi-institutional study recently published by Ohashi *et al*. [29]. There was no necrosis or sarcomatoid change in any of our cases. Based on the limited available follow-up data, it is difficult to speculate about the potential impact of the presence of the small-cell tumor component on prognosis. In no case was the aggressive behavior documented. However, the follow-up period is relatively short, with a median of 48 months.

Several neoplastic entities should be considered in differential diagnosis, especially with limited material in core biopsy, where the diagnosis may be challenging compared to the more straightforward diagnostic process in resections.

In differential diagnosis, the presence of small-cell differentiation, tubular or palisading pattern, raises the question of potential neuroendocrine differentiation (either primary or metastatic).

Primary neuroendocrine tumors of the kidney are exceedingly rare. According to the WHO classification (2016), they are subdivided into two groups: (I) Well-differentiated neuroendocrine tumor (carcinoid and atypical carcinoid) and (II) poorly differentiated neuroendocrine carcinoma including small-cell and large cell variants [12]. Morphologically, carcinoids display similar features as their counterparts in other anatomical sites. Their neuroendocrine nature is confirmed by immunohistochemistry with positive staining for neuroendocrine markers.

Ewing sarcoma/peripheral neuroendocrine tumor (PNET) must be considered in cases composed of small, round, densely packed blue cells, especially on limited material and in a young patient. PNET shows features of a highly malignant neoplasm, with numerous mitotic figures and necrosis. PNET is characterized by diffuse positivity for vimentin, CD99, and FLI-1 in immunohistochemistry. In certain cases, neuroendocrine markers may be positive [30].

In none of our cases, we found mitoses. However, in case 2, PNET was a differential diagnosis on core biopsy. On a final resection specimen, 80% of the tumor was composed of a small-cell component with PNET-like morphology, solid architecture, and densely packed cells with oval, overlapping nuclei, as well as areas with typical ChRCC morphology, haphazardly present throughout the tumor mass.

Immunohistochemical examination revealed negative staining with vimentin (typical pattern characteristic for oncocytoma), whereas FLI 1 and CD99 were negative. The morphologic characteristics of cases positive for CD99 or FLI-1 were distinct from PNET, and staining was interpreted as non-specific. None of the analyzable cases showed mutation/translocation in the EWSR gene. However, cases with non-specific FLI-1 and CD99 staining were not analyzable by NGS.

Wilms tumor (WT), blastemal-rich variant, is another example of a tumor within the spectrum of small round blue cell renal tumors. Blastema-rich WT is composed of primitive cells with sticking, highly malignant morphology showing diffuse immunoreactivity for vimentin and WT1 [12]. None of our cases showed neither such morphology nor positive staining for WT1 and/or vimentin.

The tendency of urothelial carcinoma (UC) to mimic primary renal cell carcinomas, particularly in high-grade forms, is well-known. In this regard, a macroscopic examination can give many clues for differential diagnosis. In UC, renal pelvis involvement and infiltrative growth pattern with desmoplastic response are common, whereas in ChRCC, pushing border and expansile growth pattern are more common. The infiltrative growth pattern was not reported in our study. In addition, the immunohistochemical profile of our cases differed from that of typical UC.

The final situation in differential diagnosis that should be considered is that sarcomatoid differentiation within ChRCC is relatively common. Some authors suggest that sarcomatoid dedifferentiation is more prevalent in ChRCC than in any other RCC subtype [31]. The great majority of the sarcomatoid component, on the other hand, is present in the form of a high-grade, spindle-cell, mesenchymal-looking neoplastic cell population. Necrosis is common and mitotic activity is usually brisk [32].

We were not able to identify any spindling or conspicuous mitotic figures within small cell areas, as well as necrosis. Our cases also lacked the infiltrative pattern of small cells, which would be expected in sarcomatoid dedifferentiation. The architecture and cytology of small-cell component were clearly epithelial and monotonous. All of the aforementioned characteristics argue against sarcomatoid differentiation.

## CONCLUSION

We herein present a group of 10 ChRCCs with a small-cell component that constitutes up to 80% of the tumor volume. Awareness of this unusual pattern and applying additional sections to find classic morphology of ChRCC, as well as excluding neuroendocrine nature by immunohistochemistry, may help resolve difficult cases.

However, a small-cell morphology does not present major diagnostic problem in resected tumors, on limited material, namely, as a core biopsy such morphology may create diagnostic challenge.
